# NUMT Confounding Biases Mitochondrial Heteroplasmy Calls in Favor of the Reference Allele

**DOI:** 10.3389/fcell.2019.00201

**Published:** 2019-09-25

**Authors:** Hannah Maude, Mira Davidson, Natalie Charitakis, Leo Diaz, William H. T. Bowers, Eva Gradovich, Toby Andrew, Derek Huntley

**Affiliations:** ^1^Department of Life Sciences, Imperial College London, London, United Kingdom; ^2^Section of Genomics of Common Disease, Imperial College London, London, United Kingdom

**Keywords:** nuMT, mtDNA, genotype, mitochondrial variants, mitochondrial genotype, NGS, heteroplasmy

## Abstract

Homology between mitochondrial DNA (mtDNA) and nuclear DNA of mitochondrial origin (nuMTs) causes confounding when aligning short sequence reads to the reference human genome, as the true sequence origin cannot be determined. Using a systematic *in silico* approach, we here report the impact of all potential mitochondrial variants on alignment accuracy and variant calling. A total of 49,707 possible mutations were introduced across the 16,569 bp reference mitochondrial genome (16,569 × 3 alternative alleles), one variant at-at-time. The resulting *in silico* fragmentation and alignment to the entire reference genome (GRCh38) revealed preferential mapping of mutated mitochondrial fragments to nuclear loci, as variants increased loci similarity to nuMTs, for a total of 807, 362, and 41 variants at 333, 144, and 27 positions when using 100, 150, and 300 bp single-end fragments. We subsequently modeled these affected variants at 50% heteroplasmy and carried out variant calling, observing bias in the reported allele frequencies in favor of the reference allele. Four variants (chrM:6023A, chrM:4456T, chrM:5147A, and chrM:7521A) including a possible hypertension factor, chrM:4456T, caused 100% loss of coverage at the mutated position (with all 100 bp single-end fragments aligning to homologous, nuclear positions instead of chrM), rendering these variants undetectable when aligning to the entire reference genome. Furthermore, four mitochondrial variants reported to be pathogenic were found to cause significant loss of coverage and select haplogroup-defining SNPs were shown to exacerbate the loss of coverage caused by surrounding variants. Increased fragment length and use of paired-end reads both improved alignment accuracy.

## Introduction

Accurate detection of mitochondrial mutations is important to several fields of work, including in the diagnosis of mitochondrial diseases. Since eukaryotic cells contain up to hundreds of thousands of polyploid mitochondria, several different alleles can be present at the same position in different copies of the mitochondrial genome (mtDNA); a phenomenon termed “heteroplasmy.” As a result, mitochondrial diseases are often subject to a “threshold effect,” where a pathogenic mutation must be present above a given frequency for the disease to develop. It is therefore important to accurately call allele frequency, including low-frequency mutations which can be transmitted to offspring at disease-causing frequencies or be associated with disease in their own right (Stewart and Chinnery, 2015). Complicating the accurate detection of mitochondrial variants, however, is the presence of homologous sequences within the nuclear genome resulting from the historical transfer of mitochondrial DNA (mtDNA). These “nuclear sequences of mitochondrial origin” (nuMTs) (Simone et al., 2011) have been detected at up to 1077 sites (Li et al., 2012) and may resemble up to 90% of the mitochondrial genome in length (14,904 bp) (Ramos et al., 2011).

Homology between nuMTs and mtDNA causes problems for Next Generation Sequencing (NGS) pipelines, in which short reads of DNA are aligned to the reference genome based on sequence similarity. Since the true origin of homologous reads are difficult to determine, nuMT and mtDNA cross-mapping occurs, resulting in the detection of false positive mtDNA variants (called falsely due to nuMT reads aligning to chrM) or false negatives (mtDNA variants undetected due to alignment of mtDNA reads to nuMT loci) (Albayrak et al., 2016). A popular approach when concerned with mitochondrial variants is to attempt to remove nuMTs by isolating, amplifying and sequencing the mitochondrial genome independently (Duan et al., 2018; Weerts et al., 2018). Although this is the gold-standard for genotyping mtDNA, a number of factors including the choice of reference genome still impact upon variant calls (Santibanez-Koref et al., 2018). Furthermore, large-scale diagnostic studies are increasingly turning to whole genome sequencing (WGS) in which nuclear and mtDNA are analyzed in parallel (Ouwehand, 2019). It is therefore important to further study mtDNA/nuMT confounding and to develop tools which can reliably detect mitochondrial mutations using high-throughput WGS pipelines.

This study aims to systematically investigate the accuracy of mitochondrial variant calling when aligning short DNA fragments to the whole reference human genome (GRCh38), using an *in silico* study design to report the impact of all potential mitochondrial mutations on alignment accuracy and variant calling. Allele-specific alignment of mtDNA fragments and bias in observed allele frequencies is expected at positions where variants increase sequence similarity to nuMTs.

## Materials and Methods

### Generating Artificial Mitochondrial Fragments

The reference mitochondrial genome, the Revised Cambridge Reference Sequence (rCRS, NC_012920), was downloaded and fragmented using a Python script into 100, 150, and 300 bp fragments, in sliding windows of 1 bp from starting position 1. The maximum coverage was 100×, 150× and 300×, respectively. The above was repeated for paired-end fragments and insert size was set at 200 bp for use with isaac4, giving a maximum coverage of 200×, 300×, and 600×. For bwa, the insert size was randomly selected within the range of 200–205 bp, as the algorithm required a standard deviation greater than zero to correctly align paired-end reads. Fastq files were generated and all bases were assigned a quality score of 40.

### DNA Alignment and Variant Calling

Fastq files were aligned to the reference human genome, GRCh38.p13, by means of two commonly used alignment algorithms, bwa mem [recommended by the gatk best practices pipeline (DePristo et al., 2011)] and Isaac4 (Illumina; Raczy et al., 2013). bwa mem was used with default parameters, while Isaac4 parameters were set to a maximum lane number of 1 and library sample of 1. Variant calling was carried out using mitoCaller (Ding et al., 2015).

### Simulating Mitochondrial Variants

A total of 49,707 artificially mutated mitochondrial genomes were generated, each identical to the reference rCRS except for one single nucleotide variant (SNV) (16,569 positions × three alternative alleles). Fastq files containing 100 bp, single-end fragments were created as above for each mutant genome and aligned using bwa mem. In addition, variants surrounding the linear chrM breakpoint were introduced into the shifted rCRS ± 100 bp from the breakpoint and aligned to the shifted rCRS using bwa mem (Ding et al., 2015). The % loss of coverage was calculated as the resulting coverage at the mutated position divided by the coverage at the same position following alignment of the unaltered, reference rCRS fragments. A total of 807 variants caused a coverage loss >0% when using 100 bp single-end fragments, for which alignments were repeated using 150 and 300 bp single and paired end fragments. The 807 variants were subsequently modeled at 50% heteroplasmy by concatenating the mutated rCRS fragments with the reference rCRS fragments. Datasets of 5% heteroplasmy were generated by concatenating 95 copies of the reference rCRS fragments with five copies of the mutant rCRS fragments.

### Haplotype-Defining SNPs

In order to investigate the impact on alignment of variants found in the presence of common, haplogroup-defining single-nucleotide polymorphisms (SNPs), the above was repeated for all potential variants (SNVs) within ±100 bp of 40 pre-selected haplogroup-defining SNPs. 29 SNPs defining the diverse, common haplotypes A, H, L2, M, and U, were retrieved from PhyloTree (van Oven and Kayser, 2009) (A: A1736G, A235G, A4824G, A663G, T4248C, C16290T, C8794T, G16319A, T152C, H: A2706A, C7028C, G3010A, L2: A9221G, C150T, G13590A, G16390A, G8206A, T10115C, T146C, T152C, T16311C, T2416C, M: T489C, C10400T, T14873C, G15043A, U: G12372A). An additional 11 SNPs were identified from the SNPedia list of 238 haplogroup-defining SNPs (van Oven and Kayser, 2009; Cariaso and Lennon, 2011) as causing loss of coverage in our study (100 bp, single-end fragments): C9332T, A10978G (haplogroup L6), G11914A (haplogroup C), G709A (haplogroups L6, G, N2, T), G8392A (haplogroup Y), T6221C (haplogroup X), G1888A, G13368A (haplogroup T), G8206A (haplogroup L2), G14569A (haplogroup M12’G), A5843G (haplogroup Q). An additional two SNPs, A8860G, and A4759G, were investigated, both of which are present on haplotypes other than the rCRS reference (H2a2a1) (Bandelt et al., 2014) and both of which individually caused coverage loss compared to the rCRS reference SNP (specific to H2a2a1). 100 bp single-end fragments were generated and aligned using bwa mem.

## Results

### Alignment of Reference Mitochondrial DNA to the Reference Human Genome

**FIGURE 1:**
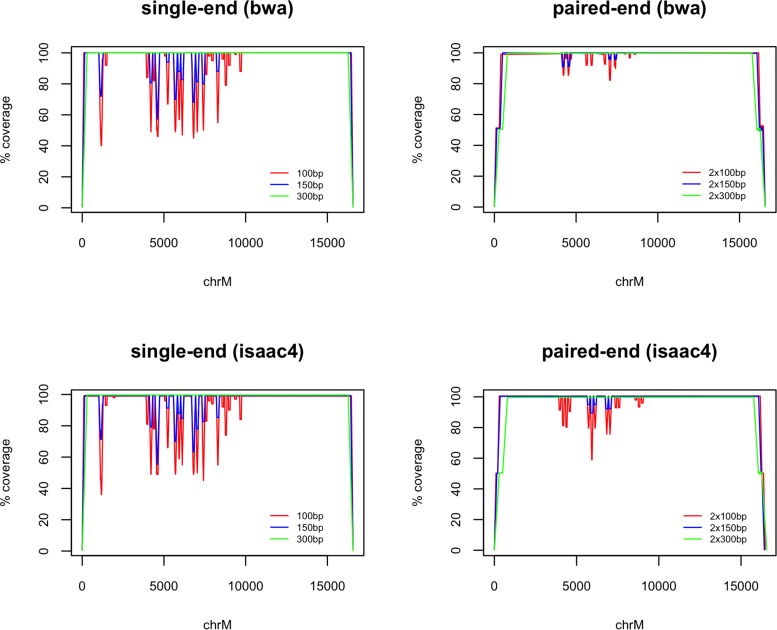
chrM coverage following whole-genome alignment of fragments generated from the reference mitochondrial genome (rCRS). The coverage is plotted as a percentage of the maximum (100×, 150×, 300× and 200×, 300×, 600× for 100 bp, 150 bp, and 300 bp single-end and paired-end fragments, respectively). Alignment was carried out using bwa mem and Isaac4. Loss of coverage is observed where fragments are assigned a primary alignment other than chrM.

Despite 100% similarity to GRCh38 chrM, alignment of mitochondrial rCRS fragments resulted in several drops in coverage where the primary alignment was not chrM (seen in Figure 1). This improved with increasing fragment length and use of paired-end fragments. There was some variation between the two alignment algorithms tested, with bwa mem performing better that isaac4 with 100 bp paired-end fragments. All 300 bp fragments aligned to chrM.

### Alignment of Mutated Mitochondrial DNA Fragments

**FIGURE 2:**
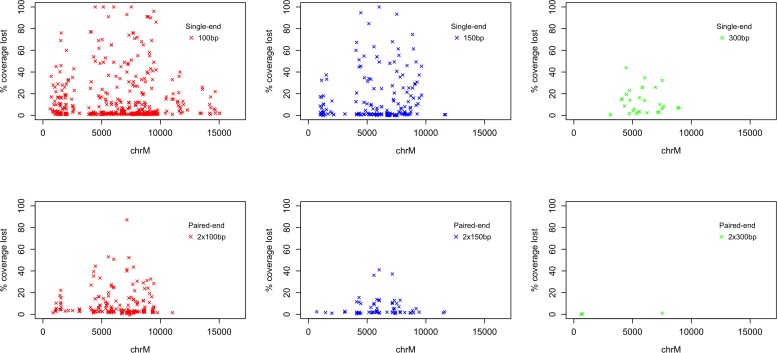
Coverage loss when aligning mitochondrial fragments containing alternative alleles. Each data point represents the alignment of the fragmented rCRS with one alternative allele (16,569 bp × 3 alternative alleles = 49,707). The % coverage loss at each mutated position is calculated as the coverage at the mutated position divided by the coverage at the same position when aligning with the reference allele. Variants which cause a loss greater than 0% using single-end fragments and greater than 3% for paired-end fragments (allowing for variation in the insert size) are plotted, allowing for up to three points per base pair position.

The coverage loss for all variants (aligned with bwa mem) is available in Supplementary Table 1 and plotted in Figure 2. A total of 807 variants at 333 positions in the mitochondrial genome caused a loss of coverage compared to the reference allele when using 100 bp single-end fragments, of which 175 caused a loss >10%, 119 caused >20%, and 39 caused >50%. Using 150 bp resulted in a loss of coverage >0% for 362 variants at 144 positions. Despite the perfect alignment of unaltered, 300bp rCRS fragments (Figure 1), a total of 41 variants at 27 positions caused a loss of >0% coverage, with 16 causing >10% and eight causing a loss of >20%. Variants which caused a coverage loss >3% when aligning paired-end fragments (to exclude variation caused by the flexible fragment size) resulted in a loss for 101, 26, and 0 variants at 61, 17, and 0 positions when using 100, 150, and 300 bp paired-end fragments, respectively.

**FIGURE 3:**
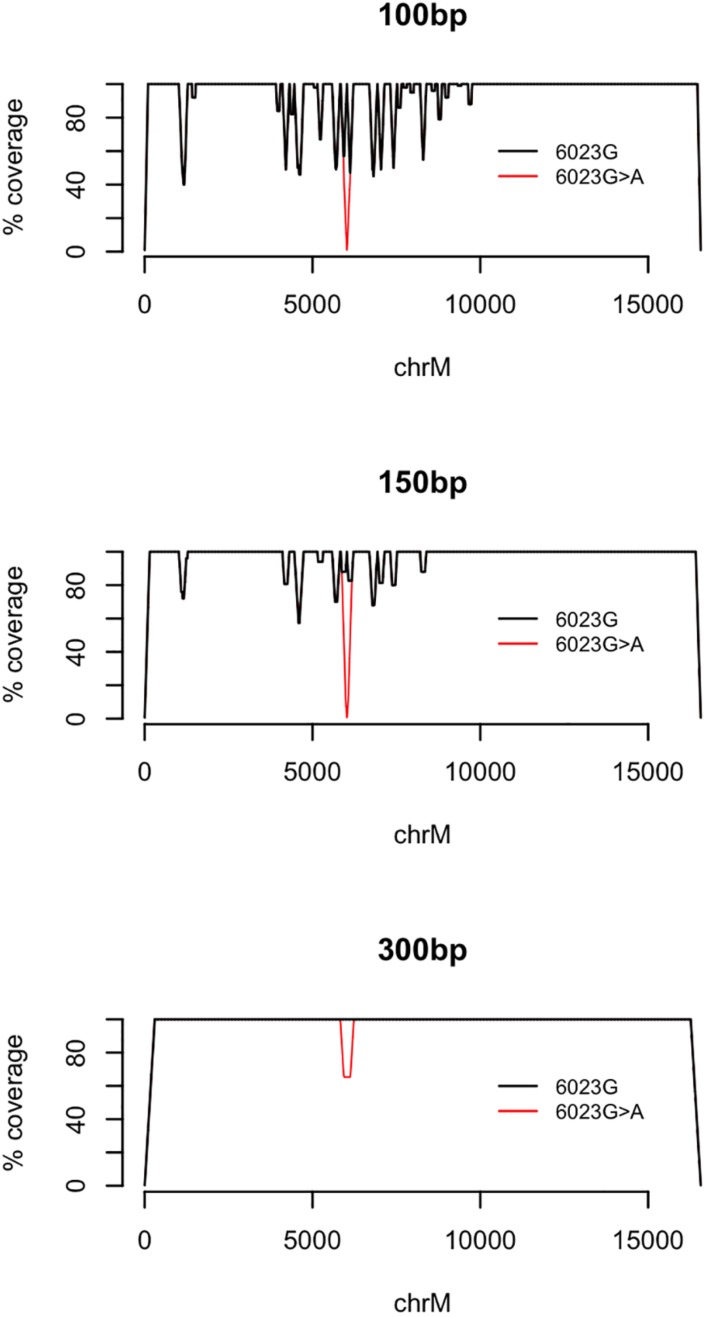
Coverage following alignment of the fragmented mitochondrial genome with the example mutation chrM:6023G > A (red) and the fragmented, unaltered rCRS mitochondrial genome (black). Alignments were carried out using the bwa mem alignment algorithm and single-end fragments of 100 bp, 150 bp, and 300 bp.

When using 100 bp single-end reads, four mitochondrial variants (G7521A, G5147A, C4456T, and C6023A) caused 100% loss of coverage, with no fragments covering the mutated position on chrM following alignment. As an example, fragments covering chrM:6023 are identical to a nuMT on chr1 with one mismatch at chr1:631,193. Introducing the alternative allele A makes the sequence 100% identical to chr1, resulting in alignment to chr1, while fragments containing the reference allele align to chrM (see Figure 3). No variants within 100 bp of the chrM breakpoint caused a loss in coverage (data not shown).

### Mutated Genome Heteroplasmy Calls

**FIGURE 4:**
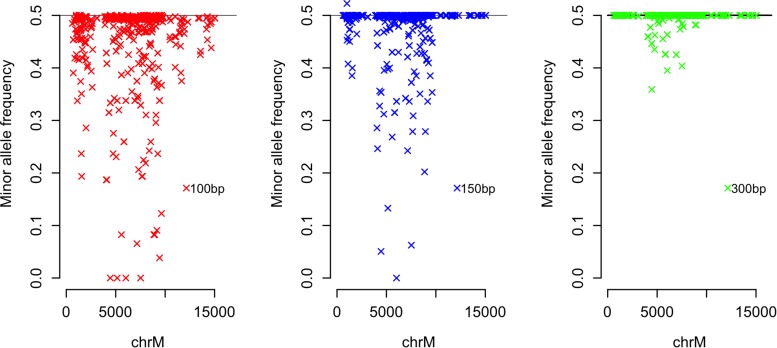
Observed minor allele frequency for mitochondrial variants modeled at 50% heteroplasmy (MAF = 0.5). Variants which cause a loss in coverage of >0% compared to the reference allele are plotted. Alignment to GRCh38 was carried out using bwa mem and variants were called using mitoCaller. The true minor allele frequency (MAF) of all variants is 0.5 although the observed MAF may be lower due to preferential alignment of fragments containing the alternative allele to nuclear loci.

The 807 variants observed to cause a loss of coverage compared to the reference allele (100 bp single-end fragments) were introduced at 50% heteroplasmy into the artificially fragmented rCRS mitochondrial genome. Figure 4 displays the observed minor allele frequencies (MAF) following alignment and variant calling (see Supplementary Table 2 for all observed MAFs when modeling 50%). Variant calls were shown to be biased in favor of the reference allele due to allele-specific alignment of mutated fragments to nuMT loci. For example, the mitochondrial variant C4456T was detected by mitoCaller at a frequency of 0, 5.1, and 35.9% when using 100, 150, and 300 bp single-end fragments, despite a true frequency of 50% (MAF = 0.5). When introduced at 5% heteroplasmy (0.05 MAF), the variant was undetected using 100 and 150 bp fragments (observed at 2.8% using 300 bp fragments), suggesting that low-frequency variants affected by nuMT confounding may be completely undetected or screened out as the observed MAF may fall below a pre-defined threshold.

### Pathogenic Variants Affected by nuMT Confounding

Three variants present in the MITOMAP database of pathogenic mitochondrial variants (Lott et al., 2014) caused a coverage loss >50%, with one additional mutation reported in the literature (A8021G). C1556T, located in the *RNR1* gene, was reported in one HCM patient (Prasad et al., 2006) and caused a coverage loss of 76% (100 bp), 33.3% (150 bp), and 0% (300 bp) when using single-end reads. G5821A, a DEAF helper mutation (Young et al., 2006; Lu et al., 2010; Levin et al., 2013) located within the *TC* gene, caused a coverage loss of 65, 54, and 26%, while A8021G (*CO2* gene) caused a loss of 72, 37.3, and 0% (100, 150, and 300 bp single-end) and also has a published link to Asthenozoospermia (Siwar et al., 2014). C4456T is a possible hypertension factor (Zhu et al., 2009) and was observed in a patient with Brugada syndrome (Tafti et al., 2018). Notably, this variant caused a total loss of 100 bp fragments (which instead aligned to chr1), rendering this mitochondrial variant undetectable when aligning reads of 100 bp or shorter to the whole reference genome. Using 150 bp resulted in a loss of 94.7% and using 300 bp resulted in a 44% loss. One additional mutation associated with the mitochondrial disease LHON (C7868T) was found to cause a loss of 10% (100 bp, single-end).

### Influence of Haplotype

The 29 SNPs representing haplogroups A, H, L2, M, and U did not cause loss of coverage, nor did their presence cause significant changes in coverage when aligning fragments containing variants within ±100 bp (results not shown). In contrast, the 11 haplogroup-defining SNPs which individually caused a loss of coverage also significantly altered the alignment of fragments in the presence of surrounding SNVs. Plots showing the loss of coverage surrounding these 11 SNPs can be seen in Supplementary Figure 1. For example, coverage dropped by 17% when the haplotype T SNP G1888A was introduced. When G1883A is introduced in conjunction with G1888A (haplotype T), 95% of fragments capturing G1883A are lost, compared to 17% when this mutation is introduced into the reference background (rCRS).

Common alternative alleles at two defining SNPs of the rCRS haplogroup H2a2a1 caused substantial coverage loss: A4769G: 62%, and A8860G: 91% (using 100 bp single-end reads). The common mutation A8860G, which is characteristic of haplogroups outside the reference H2a2a1 (Bandelt et al., 2014), caused 91% of fragments to incorrectly align to chr1, with the G allele identical to chr1:634,029. Alternative alleles within ±100 bp of these two common polymorphisms were investigated (see Supplementary Figure 1). These two SNPs exacerbated the loss of coverage for several surrounding SNVs, most notably, 99% of fragments capturing T4762C in the presence of A4769G (non-H2a2a1) were lost, compared to 67% when introduced into the rCRS (H2a2a1).

## Discussion

Sequence similarity between nuclear DNA (nuMTs) and mtDNA is a known source of confounding in NGS studies (Li et al., 2012; Albayrak et al., 2016), shown here to affect the alignment of mtDNA fragments including those identical to chrM (Figure 1). This study used an *in silico* design to document the impact of all potential single-nucleotide variants (SNVs) across the mitochondrial genome on alignment accuracy and variant calling. SNVs were introduced one at-a-time into the reference mitochondrial genome, revealing a total of 807 variants which caused preferential alignment to homologous nuMTs and biased observed allele frequencies in favor of the reference allele. The potential impact of such a “reference bias,” discussed previously (Bandelt et al., 2014) and systematically demonstrated in this study, include incorrect variants calls and the failure to detect rare variants in real data; encouraging improved data-generation and analysis.

Most of the 807 variants caused a small loss of coverage at the mutated position (chrM), however, many were associated with considerable loss, with up to 100% of mutated fragments aligning to nuMTs. These results are consistent with a previous finding by Santibanez-Koref et al. (2018) that the choice of reference genome (hg19 or rCRS, NM_012920.1) has a profound effect on the detection of mitochondrial variants (up to a 100% difference), even when analyzing isolated mtDNA. Two common alignment algorithms were here tested and shown to have negligible differences in the handling nuMT/mtDNA confounding; a comparison which can be repeated for additional bioinformatics tools. Fragment length and type was also found to influence the extent of nuMT confounding. Albayrak et al. (2016) previously suggested that single-end reads of 417 bp could be used to identify low-abundance mitochondrial mutations at 100% of locations. When using 417 bp fragments to test the two variants observed in this study that cause the largest loss in coverage, chrM:4456C > T and chrM:6023G > A, the loss at each respective position dropped to 5.5 and 0%, compared to 100% when using 100 bp fragments (data not shown), highlighting the advantage of longer read lengths.

Importantly, this study has investigated the impact of solitary SNVs on alignment accuracy. Confounding is likely to be exacerbated if multiple variants are present on the same read, demonstrated here by the worsening alignments caused by variants in the presence of common haplogroup-defining SNPs (which alignment algorithms recognize as two mismatches). To increase accuracy, NGS data should be re-aligned to a reference sequence of the correct mitochondrial haplogroup, determined by preliminary variant calling. Furthermore, removing the N at position 3107 of the reference chrM (with correction of base positions post-alignment) and aligning to a shifted genome to facilitate the circular nature of the mitochondrial genome (Ding et al., 2015) are additional best practice considerations.

It is worth noting that this study is limited to nuMTs present in the GRCh38.p13 reference genome. As additional population variation is characterized and added to the reference genome, rare, polymorphic nuMTs may present additional confounding, particularly since polymorphic nuMTs likely result from more recent mtDNA insertions and thus retain higher homology to the mtDNA. Furthermore, while this study has investigated the alignment of mitochondrial reads to nuMTs, nuMT reads can also align to chrM (Santibanez-Koref et al., 2018). To address this concerning source of error, Li et al. (2012) previously created a database of nuMTs containing mismatches from the mitochondrial genome which may appear as false heteroplasmies if aligned to chrM. Correcting for these “NUMT-affected positions” improved disparity between mitochondrial variants identified using long-range PCR vs. capture enrichment (shown to co-amplify nuMTs). Complementing this approach with additional *in silico* analysis of misaligned mitochondrial reads will ultimately enable novel tools to be developed and incorporated into WGS pipelines, improving the accuracy of mitochondrial variant calling in diagnostic and research settings.

## Data Availability Statement

All datasets generated for this study are included in the manuscript/Supplementary Files.

## Author Contributions

HM lead conception and design of the study, with contributions from DH and TA and wrote the manuscript. HM, MD, and NC carried out the *in silico* analysis, with contributions from LD, WB, and EG. All authors contributed to the manuscript revision, and read and approved the submitted version.

## Conflict of Interest

The authors declare that the research was conducted in the absence of any commercial or financial relationships that could be construed as a potential conflict of interest.
