# Supplementary material for: NUMT Confounding Biases Mitochondrial Heteroplasmy Calls in Favor of the Reference Allele
Source: Front Cell Dev Biol. 2019 Sep 25;7:201. doi: 10.3389/fcell.2019.00201 (PMC6773831; doi:10.3389/fcell.2019.00201)
Supplement: Supplementary file 1 [file Data_Sheet_1.PDF]

Supplementary Figure 1

Coverage plots for 13 Haplotype-defining variants (plus removal of chrM:3107N). Paired plots each show **left:** coverage for the +/-100bp surrounding the SNP of interest (SNP position shown by the central, vertical black line). The red line indicates the coverage when aligning 100bp single-end fragments generated from the rCRS genome (no mutations) to the reference genome, GRCh38.p13. Red crosses show the coverage at positions of SNVs which cause a loss of coverage compared to the rCRS reference allele. **Right:** the blue line shows the coverage +/-100bp surrounding the SNP of interest, when fragments containing the SNP are aligned (this represents alignment of non-mutated fragments from the relevant haplogroup, denoted in the plot title). The blue crosses show the coverage at SNV positions when each SNV is introduced into the rCRS in conjunction with the haplogroup-defining SNP.

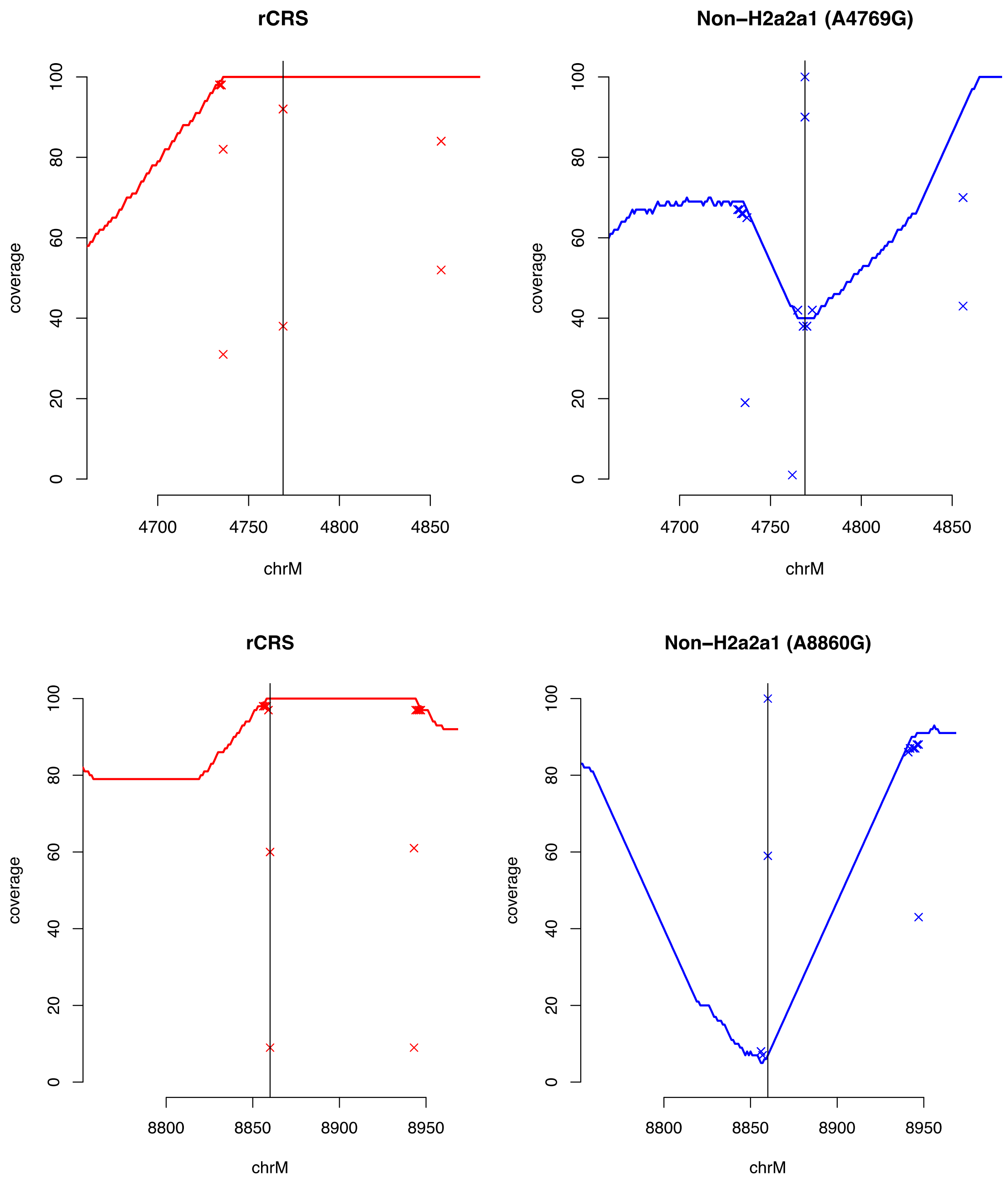

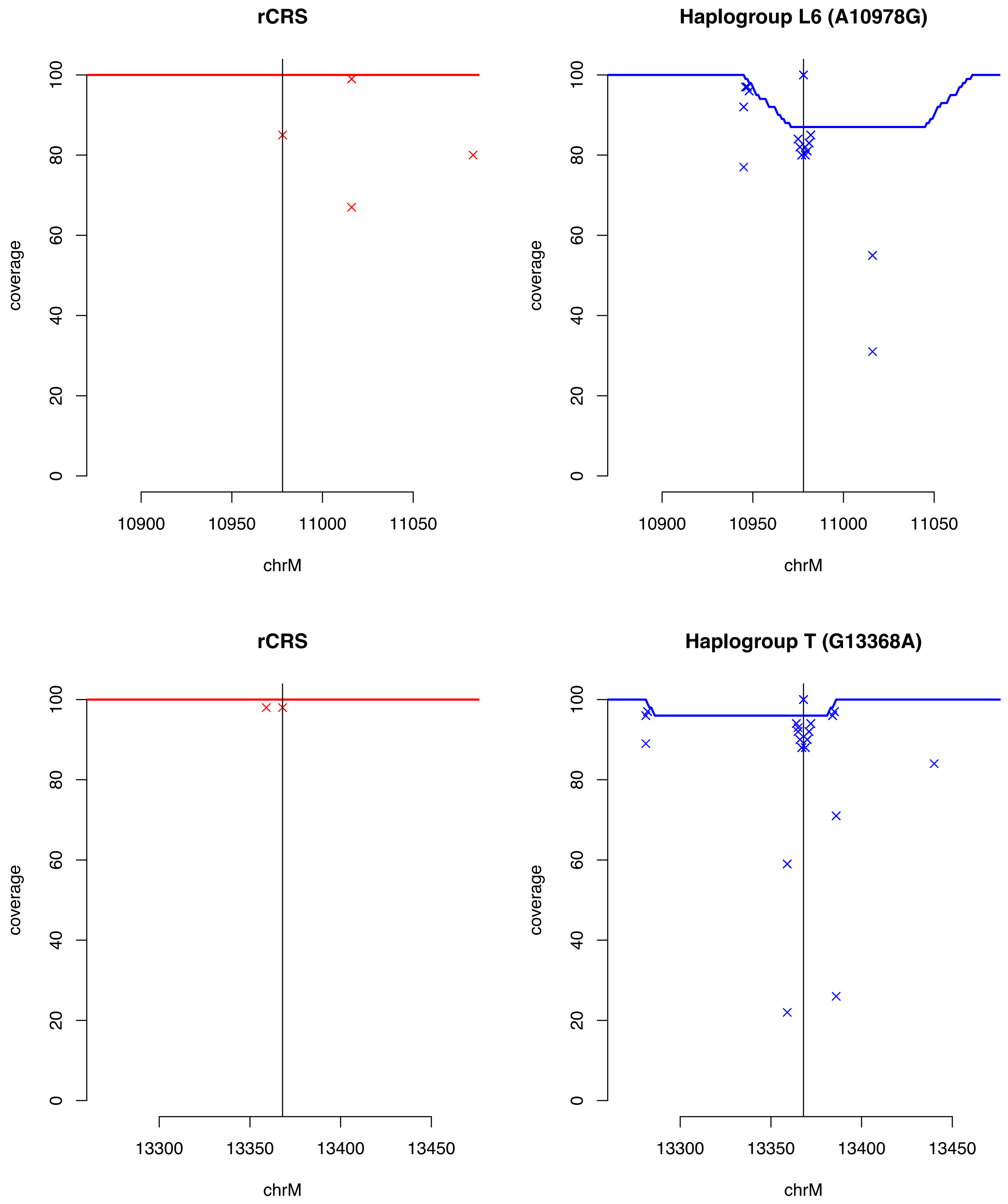
rCRS
Haplogroup L6 (A10978G)
rCRS
Haplogroup T (G13368A)
coverage
chrM
0
20
40
60
80
100
10900
10950
11000
11050
13300
13350
13400
13450

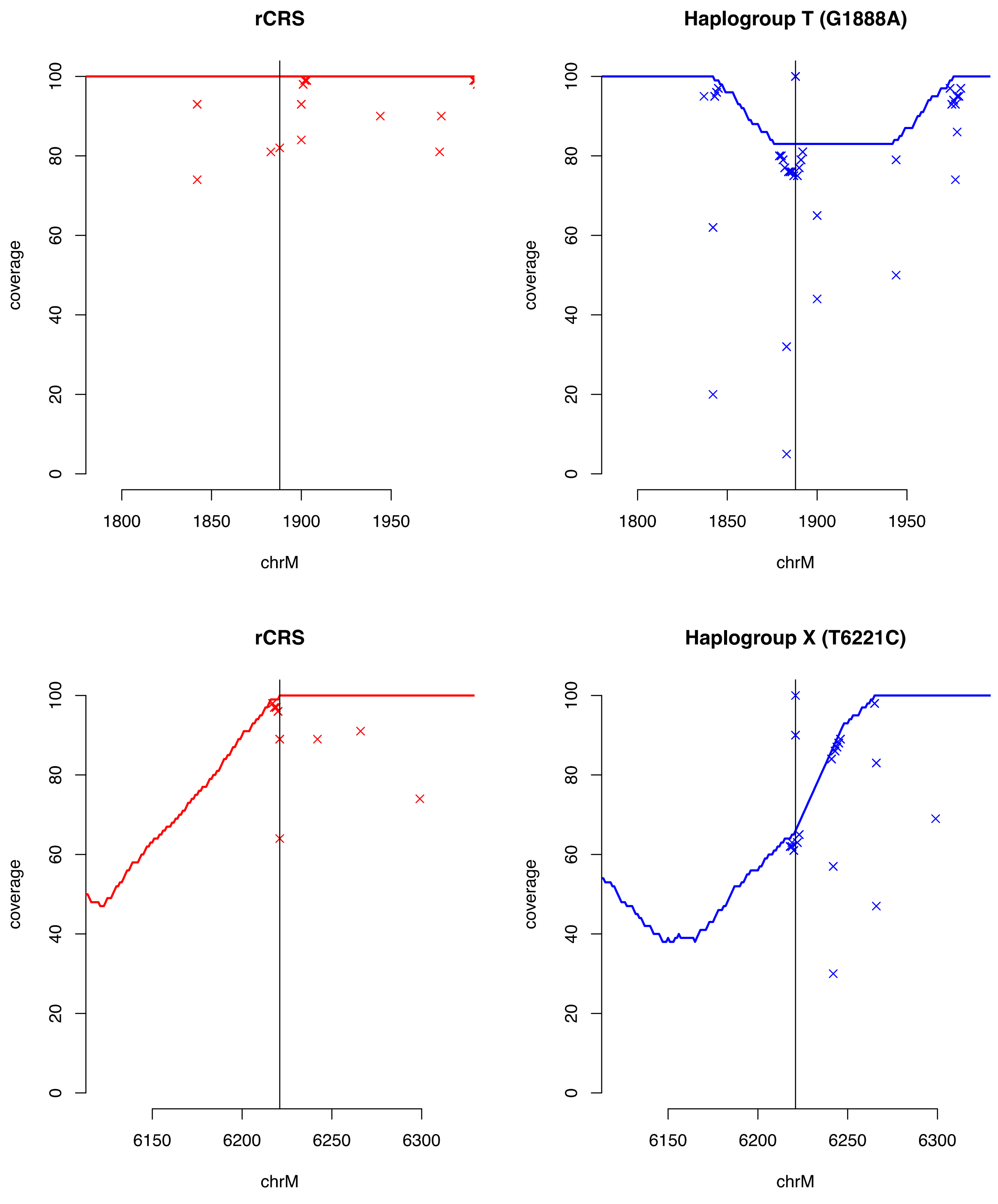
rCRS
Haplogroup T (G1888A)
rCRS
Haplogroup X (T6221C)
coverage
chrM
0
20
40
60
80
100
1800
1850
1900
1950
6150
6200
6250
6300

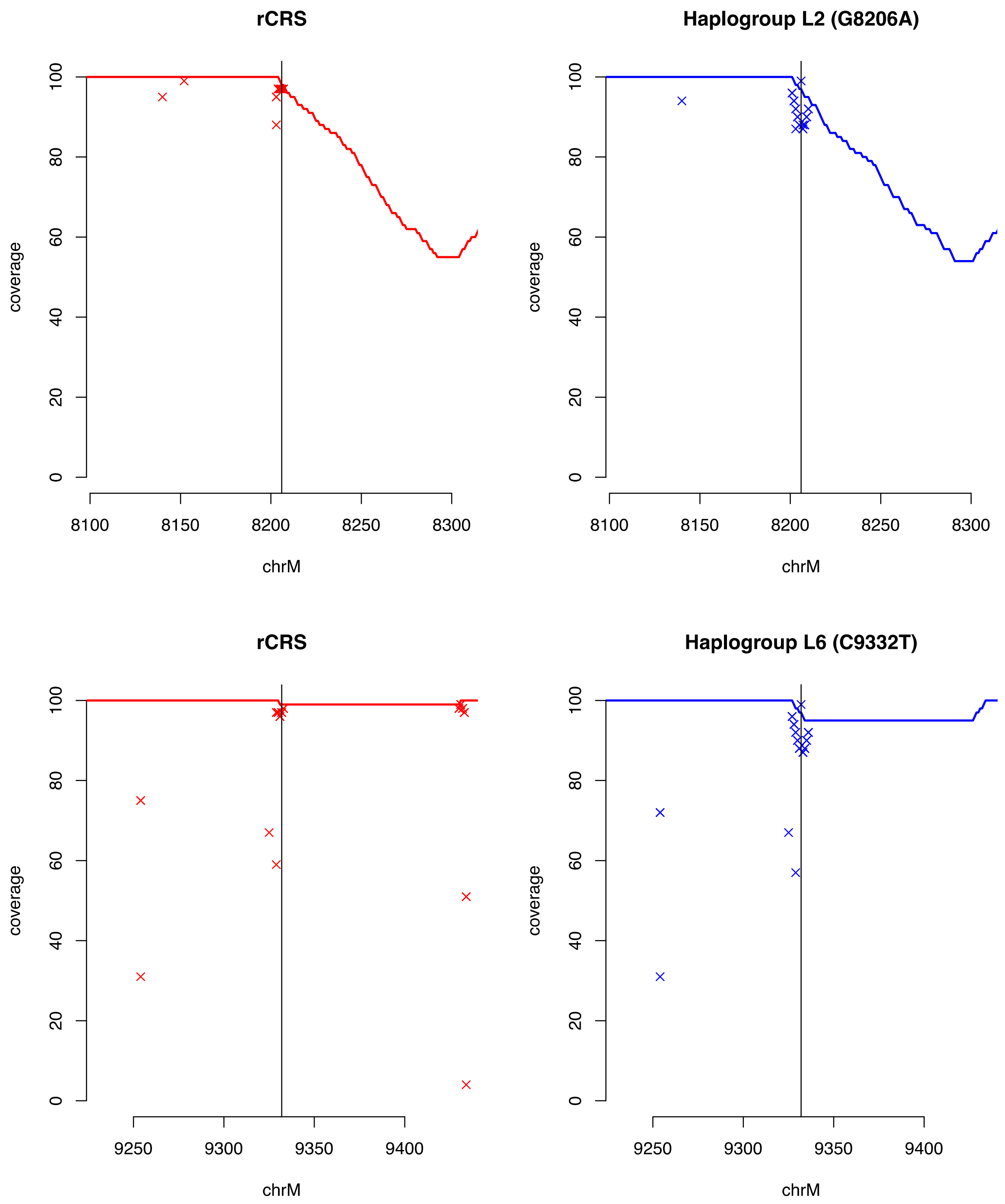

rCRS
Haplogroup L2 (G8206A)
rCRS
Haplogroup L6 (C9332T)
coverage
chrM
0
20
40
60
80
100
8100
8150
8200
8250
8300
9250
9300
9350
9400

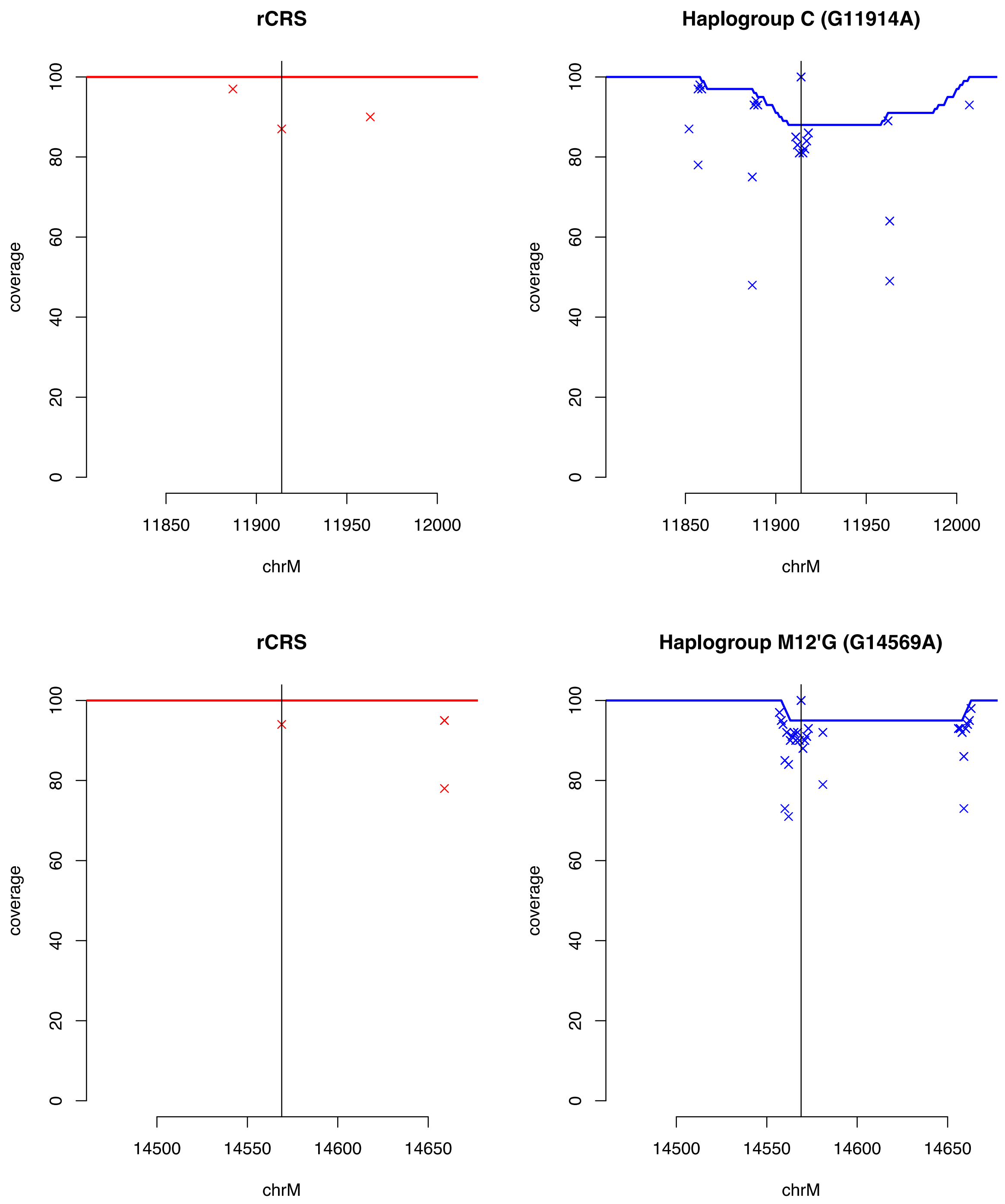
rCRS
Haplogroup C (G11914A)
rCRS
Haplogroup M12'G (G14569A)
coverage
chrM
0
20
40
60
80
100
11850
11900
11950
12000
14500
14550
14600
14650

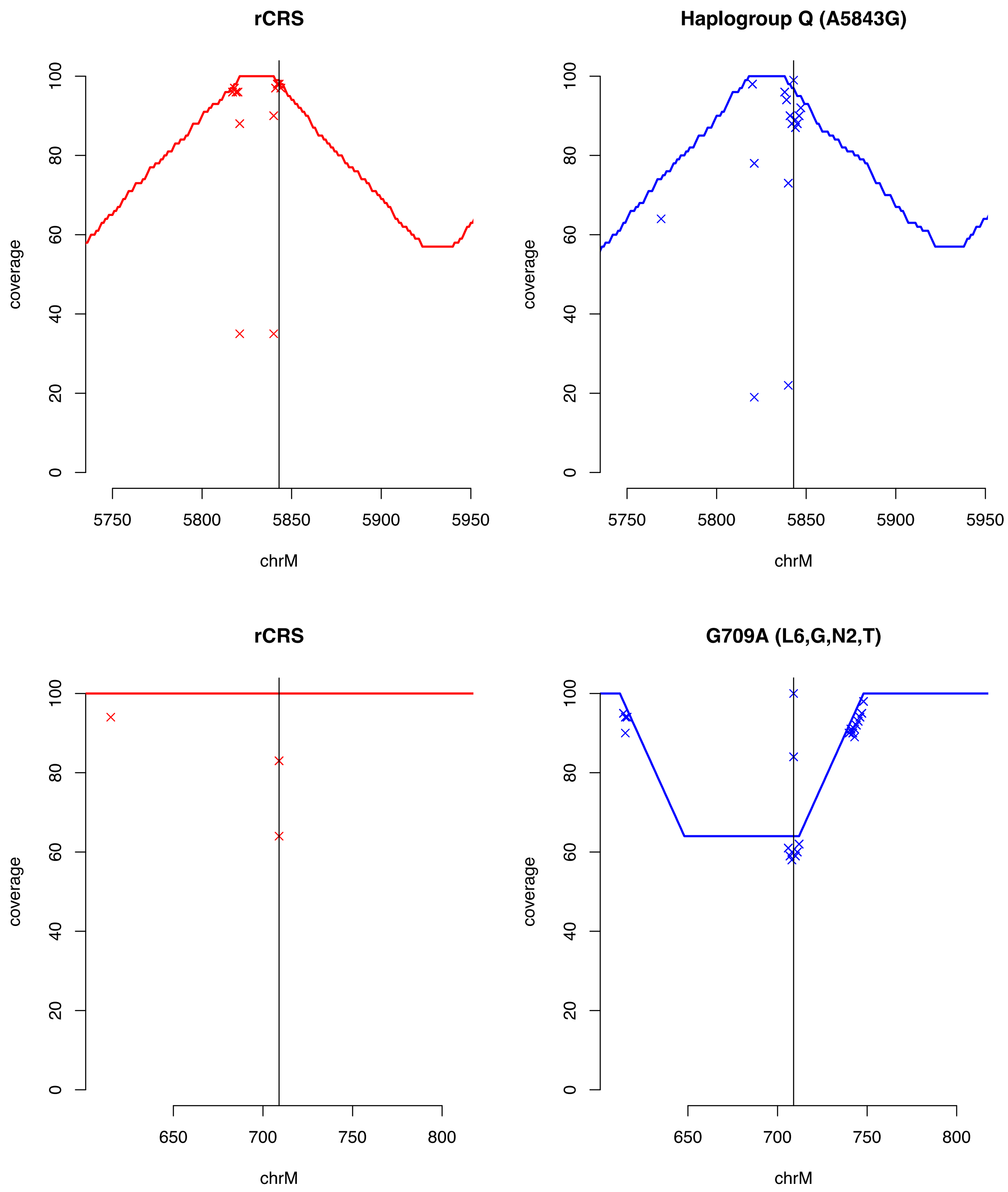
rCRS
Haplogroup Q (A5843G)
rCRS
G709A (L6,G,N2,T)
coverage
chrM
0
20
40
60
80
100
5750
5800
5850
5900
5950
650
700
750
800

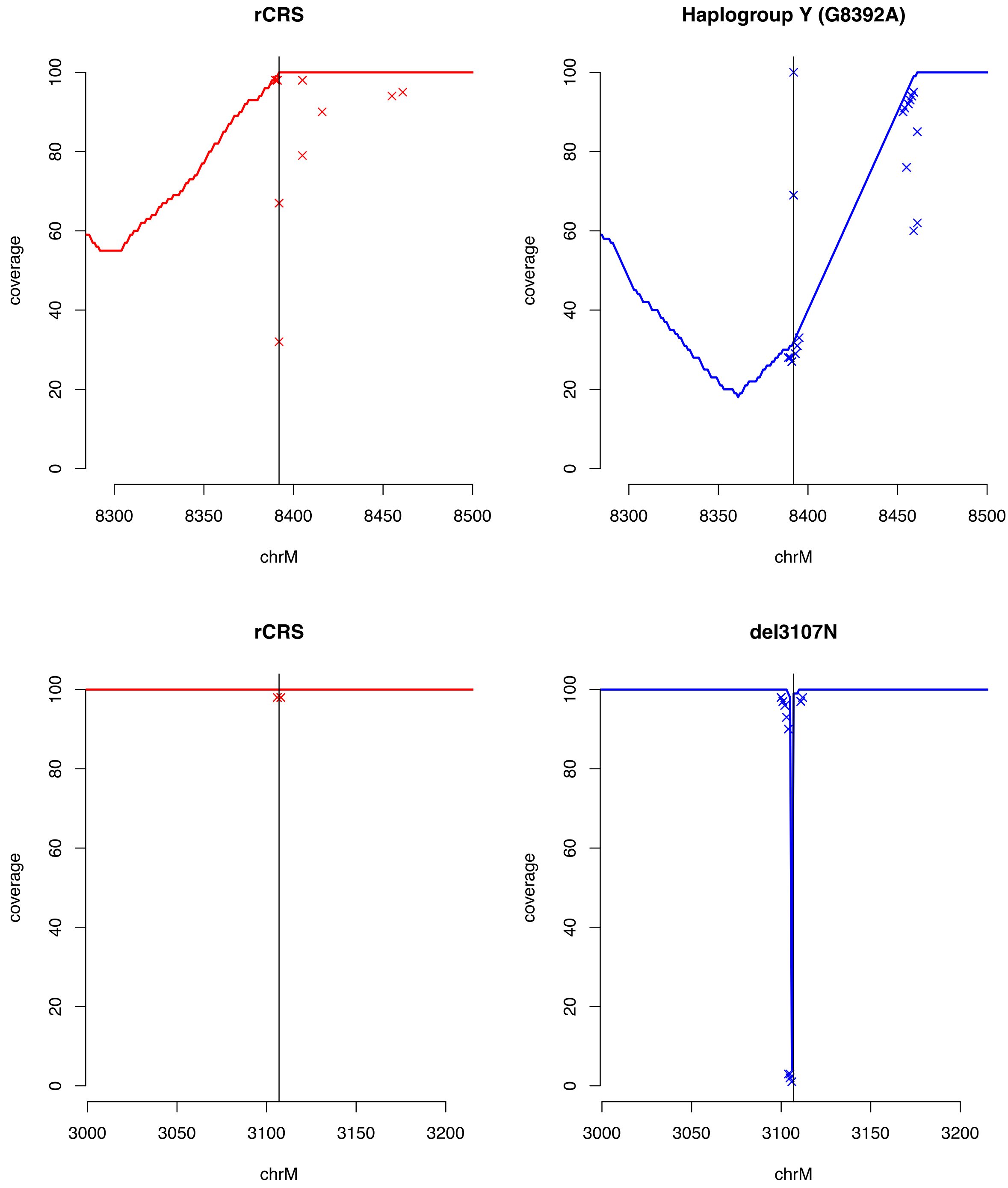

rCRS
Haplogroup Y (G8392A)
rCRS
del3107N
coverage
chrM
0
20
40
60
80
100
8300
8350
8400
8450
8500
3000
3050
3100
3150
3200
